# Generation of single-cysteine *E. coli* ProQ variants to study RNA-protein interaction mechanisms

**DOI:** 10.17912/micropub.biology.001188

**Published:** 2024-04-09

**Authors:** Helen S. Washington, Shiying Wang, Katherine E. Berry

**Affiliations:** 1 Program in Biochemistry, Mount Holyoke College, South Hadley, Massachusetts, United States; 2 Program in Biochemistry and Department of Chemistry, Mount Holyoke College, South Hadley, Massachusetts, United States

## Abstract

ProQ is a FinO-domain protein found in *E. coli* and other proteobacteria that has a global RNA-binding profile. In order to probe the detailed mechanism of RNA interactions, we have developed a collection of 13 *E. coli* ProQ variants that possess single-cysteine residues at varied positions on the surface of the N-terminal FinO domain and retain the ability to bind well to RNA. This set of variant ProQ proteins will support future biochemical and biophysical studies to map the orientation of bound RNAs to different sites around the ProQ protein, shedding light on the mechanism of ProQ-RNA interactions.

**Figure 1. Testing the RNA-binding ability of E. coli ProQ single-cysteine variants:**
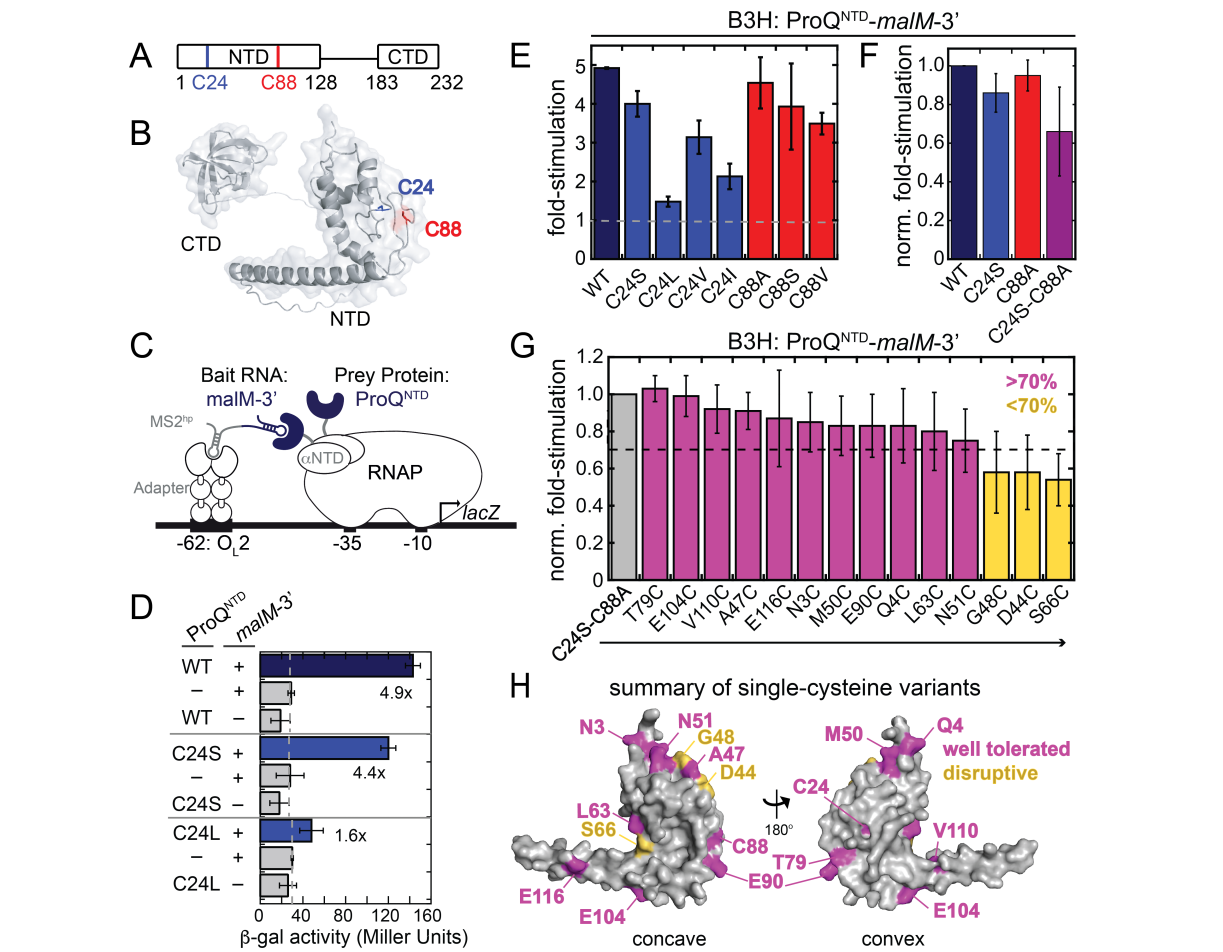
a) Domain- and b) protein-structure of *E. coli* ProQ, with the position of the two native cysteine residues highlighted. The 3D protein structure was predicted by AlphaFold (Jumper et al. 2021; Varadi et al. 2022). c) Schematic of bacterial three-hybrid (B3H) system to detect interaction between ProQ ^NTD^ and *malM* 3ʹUTR RNA, fused to the NTD of the α subunit of RNAP and an MS2 RNA hairpin (MS2 ^hp^ ; see methods), respectively. d-f) Results of β-galactosidase assays testing the interaction between ProQ ^NTD^ and *malM-* 3’UTR using the B3H assay (Stockert et al. 2022; Pandey et al. 2020). The interactions of ProQ ^NTD^ mutants lacking one or both native cysteine residues are compared to wild-type (WT) ProQ. Data are plotted with varying levels of normalization: d) raw β-galactosidase data are shown for each transformation of KB473 reporter cells with pPrey-ProQ ^NTD^ (pKB951 or a mutant derivative) and pBait- *malM* -3’ (pKB1210) or the corresponding negative controls (alpha empty and pCH1, respectively). The fold-stimulation over basal levels of *lacZ* activity is defined as the ratio of *lacZ* activity when both bait and prey are present divided by the highest value from the negative controls when either bait or prey is missing. e) B3H data of additional single-cysteine ProQ ^NTD^ mutants are plotted as fold-stimulation over basal levels of *lacZ* activity. f) B3H data of single and double-cysteine ProQ ^NTD^ mutants are plotted as normalized fold-stimulation values, where the fold-stimulation of ProQ ^WT^ is set to 1.0 and a fold-stimulation of 1.0, representing no B3H interaction, is set to 0 (see Methods). g) B3H data comparing interaction of ProQ ^C24S-C88A^ to versions of this cysteine-free ProQ ^NTD^ containing additional mutations introducing single cysteine residues. Data are plotted as normalized fold-stimulation values, where the fold-stimulation of ProQ ^C24S-C88A^ is set to 1.0. Data from mutants producing at least 70% of the interaction of ProQ ^C24S-C88A^ are shown in magenta and those <70% are shown in yellow. In c-f), bar graphs show the average and standard deviations of values collected from three or more independent experiments conducted across multiple days. h) Locations of residues substituted with single-cysteine mutations are shown on the AlphaFold-predicted structure (Jumper et al. 2021; Varadi et al. 2022); residues are colored as in (g) based on the strength of RNA-binding of the ProQ ^NTD^ variant with a cysteine at each position.

## Description

RNA-binding proteins (RBPs) play essential roles in gene regulation in all organisms, including bacteria. Hfq is the most well characterized bacterial RNA-chaperone protein that stabilizes small RNAs (sRNAs) and facilitates sRNA base pairing with 5’ untranslated regions (UTRs) of mRNAs. One class of bacterial RBPs that has garnered recent attention is FinO-domain proteins (Liao and Smirnov 2023) ; the *E. coli* ProQ protein is a global RBP with many sRNA and mRNA targets (Olejniczak and Storz 2017; Holmqvist et al. 2020) (Fig 1a,b). The core RNA-binding site of ProQ is found in a conserved concave pocket in the FinO-like N-terminal domain (NTD), which recognizes the 3’ base and poly(U) tail of intrinsic terminators (Kim et al. 2022; Stein et al. 2023; Pandey et al. 2020) . Still, several important questions remain unanswered about how ProQ interacts with RNA, such as: what role the C-terminal domain (CTD) and/or unstructured linker of *E. coli* ProQ play in RNA interactions; the degree of conformational flexibility and heterogeneity of ProQ-RNA interactions; whether RNA upstream of the intrinsic terminator participates in transient protein interactions; which surfaces ProQ uses to interact with RNA ligands that do not possess a 3’ intrinsic terminator, such as fragments of 5’ UTRs or open-reading frames (ORFs) (Holmqvist et al. 2018; Melamed et al. 2020) ; and whether ProQ can interact with two RNAs simultaneously or otherwise promote restructuring of RNAs. These mechanistic questions are important to fully understanding the role ProQ plays in regulating RNA stability and gene expression.

Several biochemical and biophysical techniques make use of site-directed bioconjugation of macromolecules for functionalization with small-molecule ligands (Hermanson 2013) . For instance, site-specific hydroxyl radical probing with an FeBABE reagent can reveal details of RNA-protein interaction mechanisms (Duval et al. 2017) . This approach makes use of the uniquely nucleophilic thiol group of cysteine side-chains (Gunnoo and Madder 2016) : single-cysteine variants of a protein of interest are achieved by the removal of surface-exposed native cysteines and the installation of new cysteine residues that can be site-specifically labeled with a thiol-reactive FeBABE molecule (Duval et al. 2017) . An important step in this process is identifying locations of cysteine variants that do not interfere with the native protein’s function. In order to identify single-cysteine variants of *E. coli* ProQ that retain the ability to bind to RNA targets, we utilized a bacterial three-hybrid assay (B3H; (Berry and Hochschild 2018; Stockert et al. 2022) to test the RNA-binding activity of a panel of cysteine-free and single-cysteine variants of this protein. Briefly, this genetic assay makes use of a *lacZ* reporter gene where transcription is increased by interaction between a ‘bait’ RNA moiety and a ‘prey’ protein of interest (Fig 1c; see methods).

*E. coli* ProQ naturally possesses two cysteine residues—both in the N-terminal FinO domain (Fig 1a,b). We began by mutating each of these cysteine codons to either hydrophilic or hydrophobic amino acids ( *e.g.* serine, leucine, valine or alanine) and testing their interactions in a B3H assay with the *malM* 3’ UTR (Fig 1d,e) *.* This RNA has been previously identified as a ProQ interactor in CLIP-seq and RIL-seq experiments (Holmqvist et al. 2018; Melamed et al. 2020) and its interaction with ProQ has been previously characterized in the B3H assay (Stein et al. 2020, 2023) . In the B3H analysis of ProQ binding to *malM-3’* , Cys24 was most robustly replaced by a serine residue (C24S), while Cys88 was best replaced by an alanine (C88A). A cysteine-free ProQ containing both of these substitutions together (C24S-C88A) retained ~70% of WT ProQ’s interaction with *malM-3’* (Fig 1f) *.* Using this cysteine-free C24S-C88A variant, we selected 14 additional residues that were predicted to be surface-exposed and not highly conserved to mutate to cysteine. These single-cysteine variants were tested for *malM-3’* interaction (Fig 1g). While some cysteine mutants were strongly disruptive to RNA interaction ( *e.g.* G48C, D44C, S66C), 11 mutants retained at least 70% of the cysteine-free variant’s interaction and were deemed suitable for future purification and site-directed conjugation, along with the C88A and C24S single-cysteine proteins. Importantly, this collection of 13 single-cysteine mutants encircles both the concave and convex faces of ProQ (Fig 1h).

Because the interaction of 3’ UTRs with ProQ does not depend strongly on the CTD or unstructured linker (Pandey et al. 2020; Stein et al. 2020) , we were unable to test single-cysteine mutants in these regions. However, it should be possible to introduce cysteine residues into positions of limited conservation that are predicted to be surface-exposed. One caveat to keep in mind is that ProQ variants may be less stable *in vitro* than they are when expressed inside of the cell as fusion proteins with α-NTD. It is also possible that a cysteine would be tolerated at a given position but that a larger moiety such as FeBABE or a fluorophore would sterically disrupt interaction. The RNA binding of site-specifically labeled proteins should therefore be confirmed under the conditions used for downstream biochemical or biophysical experiments.

## Methods

**Bacterial Strains and Plasmids.** Plasmids, oligonucleotides and bacterial strains used in this study are provided in the Reagents section below. Site-directed mutants were constructed using Q5 site-directed mutagenesis (New England Biolabs) as previously described (Stein et al. 2023) with the specified mutagenic end-to-end primers, designed with NEBaseChanger.

**β-galactosidase assays.** RNA binding of ProQ mutants were assessed using a previously established bacterial three-hybrid (B3H) assay (Fig 1c) (Stockert et al. 2022; Berry and Hochschild 2018) . Bait RNA is anchored to the B3H system by a DNA-RNA ‘adapter’ protein that consists of a fusion between the CI protein from bacteriophage 𝜆 and the bacteriophage MS2 coat protein. Bait RNA is fused to an MS2 RNA hairpin (MS2 ^hp^ ). The prey protein is fused to the NTD of the RNA polymerase (RNAP) alpha subunit (α). Interaction between the bait RNA and prey protein is measured by the fold-stimulation of *lacZ* activity when both bait and prey are present in reporter cells over basal levels, defined by the highest value of negative controls when either bait or prey protein is missing.

For B3H assays, Δ *hfq* p *lac* -O _L_ 2–62 reporter strain cells (KB473) were transformed with three compatible plasmids: first by pAdapter (pCW17), and subsequently with a combination pPrey and pBait plasmids. pPrey plasmids encoded either the WT α-ProQ ^NTD^ fusion protein (resi=2-131; pKB951 or a site-specific mutant derivative) or α alone as a negative control. pBait plasmids encoded a hybrid RNA with the 3’ UTR of *malM* following the MS2 ^hp^ (pKB1210) or an RNA that contained only the MS2 ^hp^ moiety (pCH1) as a negative control. Transformations were conducted in 96-well plates as previously described (Stockert et al. 2022) . Overnight cultures were directly inoculated with transformants and grown in 1 mL of LB supplemented with carbenicillin (100 μg/mL), chloramphenicol (25 μg/mL), kanamycin (50 μg/mL), spectinomycin (100 μg/mL) and 0.2% arabinose. Plates were sealed with breathable film and grown ON at 37℃, shaking at 900 rpm. In the morning, day cultures (200 μL) were inoculated with 10 μL of ON culture, covered with a plastic lid and grown at 37℃, shaking at 900 rpm until OD _600_ values reached mid-log (0.3-0.6). Cells were lysed with the addition of 10 μL of PopCulture Reagent (Novagen) and of 4 units of rLysozyme (Novagen). β-gal activity was measured as previously described (Stockert et al. 2022) .

The β-gal activity produced by reporter cells transformed with each combination of bait RNA and prey protein was divided by the highest value of the two corresponding negative controls to yield fold-stimulation over basal *lacZ* levels. These fold-stimulation values were then normalized using the equation: (fold-stimulation ^mutant^ -1)/(fold-stimulation ^comparison^ -1), where the point of comparison was either the fold-stimulation produced by ProQ ^WT^ or ProQ ^C24S-C88A^ . This normalization sets the B3H value for the highest value to 1.0 and the lack of a detectable interaction (1-fold-stimulation of *lacZ)* equal to 0.

**Protein Structures.** The positions of native and introduced cysteine residues were mapped onto the AlphaFold predicted structure of *E. coli* ProQ (Jumper et al. 2021; Varadi et al. 2022) , which has been found to be consistent with genetic mutagenesis data (Stein et al. 2023) . Structures were rendered in the PyMOL Molecular Graphics System (Schrödinger, LLC).

## Reagents

**Plasmids used in this study:**

**Table:** 

**Name**	**Description**	**Details**	**Reference/ Oligos used in this study**
pAClCI	empty vector	Encodes full-length lCI under the control of the *lacUV5* promoter; confers CamR	(Dove et al. 1997)
pBrα	Empty vector	Encodes full-length *rpoA* under the control of tandem *lpp* and *lacUV5* promoters; confers AmpR	(Dove et al. 1997)
pCH1	pCDF-1XMS2hp (empty vector)	pCDF-pBAD-MS2hp-XmaI-HindIII; confers SpcR	(Pandey et al., 2020)
pCW17	pAC-constitutive-λCIMS2CP	Encodes residues 1-248 of CI fused to an MS2 coat protein; transcription of this protein under the control of a constitutive promoter; confers CmR	(Pandey et al., 2020)
pKB951	pBrα-ProQ ^NTD^	Encodes residues 1-248 of alpha fused to residues 1-131 of wild type *E. coli proQ* ; TAA stop codon after *proQ* added through PCR; confers AmpR	(Pandey et al., 2020)
pKB1210	pCDF-pBAD-1xMS2- *malM* -3’UTR	3'UTR of *E. coli malM* (final 90 nts) cloned behind MS2hp in pCH1 between XmaI/ HindIII sites; RNA encodes its own terminator; confers SpcR	(Stein et al. 2020)
pASW24	pBrα-ProQ ^NTD^ -C24S	*proQ* C24S mutation introduced to pKB951	oASW55 + oASW56
pASW25	pBrα-ProQ ^NTD^ -C24L	*proQ* C24L mutation introduced to pKB951	oKB1552 + oKB1553
pASW26	pBrα-ProQ ^NTD^ -C24V	*proQ* C24V mutation introduced to pKB951	oKB1552 + oKB1553
pASW27	pBrα-ProQ ^NTD^ -C24I	*proQ* C24I mutation introduced to pKB951	oKB1552 + oKB1553
pASW28	pBrα-ProQ ^NTD^ -C88A	*proQ* C88A mutation introduced to pKB951	oASW53 + oASW54
pASW29	pBrα-ProQ ^NTD^ -C88S	*proQ* C88S mutation introduced to pKB951	oASW31 + oASW32
pASW30	pBrα-ProQ ^NTD^ -C88V	*proQ* C88V mutation introduced to pKB951	oASW33 + oASW34
pASW35	pBrα-ProQ ^NTD^ -C24S-C88A	*proQ* C24S mutation introduced to pASW28 (C88A)	oASW55 + oASW56
pASW39	pBrα-ProQ ^NTD^ -C24S-C88A-T79C	*proQ* T79C mutation introduced to pASW35 (C24S-C88A)	oASW65 + oASW66
pASW43	pBrα-ProQ ^NTD^ -C24S-C88A-M50C	*proQ* M50C mutation introduced to pASW35 (C24S-C88A)	oASW73 + oASW74
pASW44	pBrα-ProQ ^NTD^ -C24S-C88A-E116C	*proQ* E116C mutation introduced to pASW35 (C24S-C88A)	oASW75 + oASW76
pASW46	pBrα-ProQ ^NTD^ -C24S-C88A-E90C	*proQ* E90C mutation introduced to pASW35 (C24S-C88A)	oASW80 + oASW89
pASW47	pBrα-ProQ ^NTD^ -C24S-C88A-L63C	*proQ* L63C mutation introduced to pASW35 (C24S-C88A)	oASW81 + oASW82
pASW48	pBrα-ProQ ^NTD^ -C24S-C88A-A47C	*proQ* A47C mutation introduced to pASW35 (C24S-C88A)	oASW83 + oASW84
pASW49	pBrα-ProQ ^NTD^ -C24S-C88A-N51C	*proQ* N51C mutation introduced to pASW35 (C24S-C88A)	oASW85 + oASW86
pHW1	pBrα-ProQ ^NTD^ -C24S-C88A-N3C	*proQ* N3C mutation introduced to pASW35 (C24S-C88A)	oHW1 + oHW2
pHW2	pBrα-ProQ ^NTD^ -C24S-C88A-Q4C	*proQ* Q4C mutation introduced to pASW35 (C24S-C88A)	oHW3 + oHW4
pHW3	pBrα-ProQ ^NTD^ -C24S-C88A-D44C	*proQ* D44C mutation introduced to pASW35 (C24S-C88A)	oHW5 + oHW6
pHW4	pBrα-ProQ ^NTD^ -C24S-C88A-G48C	*proQ* G48C mutation introduced to pASW35 (C24S-C88A)	oHW7 + oHW8
pHW5	pBrα-ProQ ^NTD^ -C24S-C88A-S66C	*proQ* S66C mutation introduced to pASW35 (C24S-C88A)	oHW9 + oHW10
pHW7	pBrα-ProQ ^NTD^ -C24S-C88A-E104C	*proQ* E104C mutation introduced to pASW35 (C24S-C88A)	oHW13 + oHW14
pHW8	pBrα-ProQ ^NTD^ -C24S-C88A-V110C	*proQ* V110C mutation introduced to pASW35 (C24S-C88A)	oHW15 + oHW16

**Oligonucleotides used in this study:**

**Table:** 

**Name**	**Used For**	**Sequence**
oASW31	Q5 F C88S	CGGCAACCCAaGCGGTGAGCT
oASW32	Q5 R C88S	TCAAGATCGACACGCGTTGC
oASW33	Q5 F C88V	CGGCAACCCAgtCGGTGAGCTG
oASW34	Q5 R C88V	TCAAGATCGACACGCGTT
oASW53	Q5 F C88A	CGGCAACCCAgcCGGTGAGCTG
oASW54	Q5 R C88A	TCAAGATCGACACGCGTTG
oASW55	Q5 F C24S	TTTCCCCACTcTTTCAGTGCGGAAG
oASW56	Q5 R C24S	ACGTTCGGCCAGAAACGC
oASW65	Q5 F T79C	ACCCGGCGCAtgtCGTGTCGATC
oASW66	Q5 R T79C	TTAACACCGTAAAGATAACG
oASW73	Q5 F M50C	TGCTGGGGAAtgcAACCTGAGCAAAAC
oASW74	Q5 R M50C	ACACGATCGACCAAATCC
oASW75	Q5 F E116C	ACAGCGTGCTtgtCAGCAAGCGAAAAAAC
oASW76	Q5 R E116C	GCCTGAACACGCGCTTTC
oASW89	Q5 F C88A-E90C	CCCAgccGGTtgtCTGGACGAGC
oASW80	Q5 R C88A-E90C	TTGCCGTCAAGATCGACAC
oASW81	Q5 F L63C	CGCTTTACGTtgcTACACTTCGAG
oASW82	Q5 R L63C	GATCGCAATTGCGTTTTG
oASW83	Q5 F A47C	CGATCGTGTTtgtGGGGAAATGAAC
oASW84	Q5 R A47C	ACCAAATCCTGAAAAATACC
oASW85	Q5 F N51C	TGGGGAAATGtgcCTGAGCAAAAC
oASW86	Q5 R N51C	GCAACACGATCGACCAAA
oHW1	Q5 F N3C	GGCCGCAGAAtgTCAACCTAAGTTG
oHW2	Q5 R N3C	GCCTCTGGTTTCTCTTCTTTC
oHW3	Q5 F Q4C	CGCAGAAAATtgcCCTAAGTTGAATAGCAGTAAAG
oHW4	Q5 R Q4C	GCCGCCTCTGGTTTCTCT
oHW5	Q5 F D44C	GGATTTGGTCtgTCGTGTTGCTGG
oHW6	Q5 R D44C	TGAAAAATACCGATTTTCAG
oHW7	Q5 F G48C	TCGTGTTGCTtgcGAAATGAACCTG
oHW8	Q5 R G48C	TCGACCAAATCCTGAAAAATAC
oHW9	Q5 F S66C	CTCTACACTTgcAGCTGGCGTTATC
oHW10	Q5 R S66C	ACGTAAAGCGGATCGCAA
oHW13	Q5 F E104C	CAAGCAGCTTtgcGAAGCGAAAGC
oHW14	Q5 R E104C	CGAGCATGCTCTACATGTTG
oHW15	Q5 F V110C	GAAAGCGCGTtgTCAGGCACAG
oHW16	Q5 R V110C	GCTTCTTCAAGCTGCTTG
oKB1552	Q5 F C24L/V/I	TTTTCCCCACntaTTCAGTGCGGAAGGTGAAGCG (degenerate codon encodes each of 3 amino acids; individual colonies were sequenced to clone each individual mutant)
oKB1553	Q5 R C24L/V/I	CGTTCGGCCAGAAACGCG

**Bacterial strains used in this study:**

**Table:** 

**Name**	**Details**	**Antibiotic Resistance**	**Reference/Source**
NEB 5α F’Iq	*E. coli lacIq* host strain for plasmid construction	TetR	New England Biolabs
KB473	FW102 *∆hfq* cells with an F’episome which has test promoter placO _L_ 2-62 fused to *lacZ*	KanR, StrR	(Berry & Hochschild, 2018)
